# Supplementary material for: Aphid Resistance Evaluation and Constitutive Resistance Analysis of Eighteen Lilies
Source: Insects. 2023 Dec 8;14(12):936. doi: 10.3390/insects14120936 (PMC10743574; doi:10.3390/insects14120936)
Supplement: Supplementary file 1 [file insects-14-00936-s001.zip › insects-2715270-supplementary.pdf]

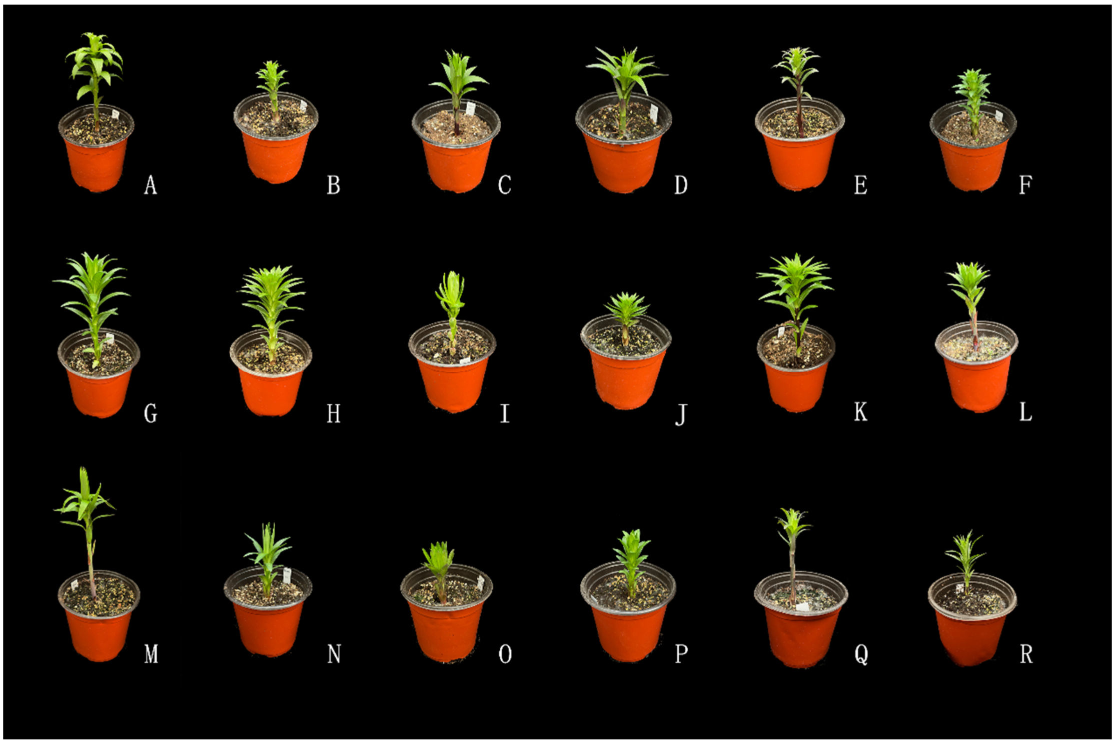

Figure S1. The growth of eighteen lilies during aphid inoculation. (A) 'Black Beauty'; (B) 'Conca D'or'; (C) 'Palazzo'; (D) 'Nymph'; (E) 'Friso'; (F) 'Eyeliner'; (G) 'Armandale'; (H) 'Heartstrings'; (I) 'Apricot Fudge'; (J) 'Trendy Havana'; (K) 'Secret Kiss'; (L) 'Cameleon'; (M) 'The Edge'; (N) 'White Triump'; (O) 'Magnefique'; (P) 'Watch Up'; (Q) *Lilium leucanthum*; (R) *Lilium lancifolium*.

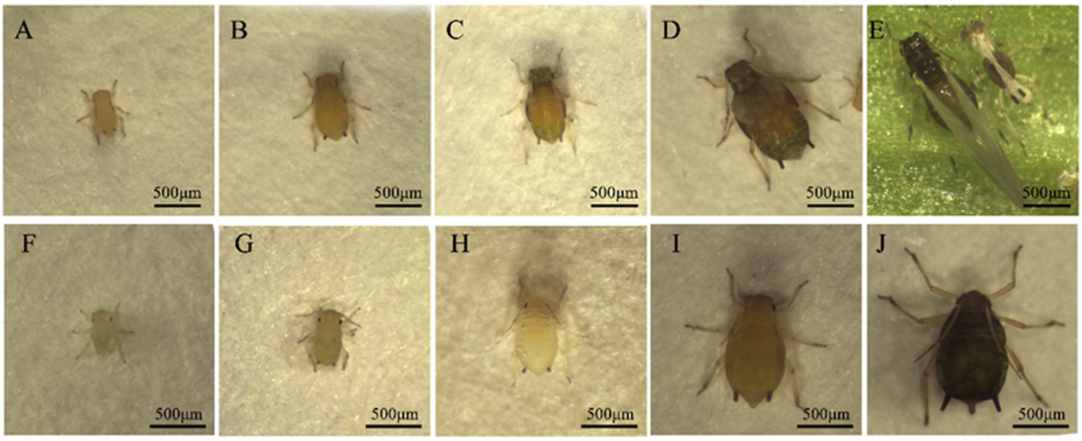

Figure S2. Comparison of body form of various ages and wing morphs of *Aphis gossypii*. (A) 1st instar alate nymph; (B) 2nd instar alate nymph; (C) 3rd instar alate nymph; (D) 4th instar alate nymph; (E) Alate adult; (F) 1st instar apterous nymph; (G) 2nd instar apterous nymph; (H) 3rd instar alate apterous nymph; (I) 4th instar apterous nymph; (J) Apterous adult.

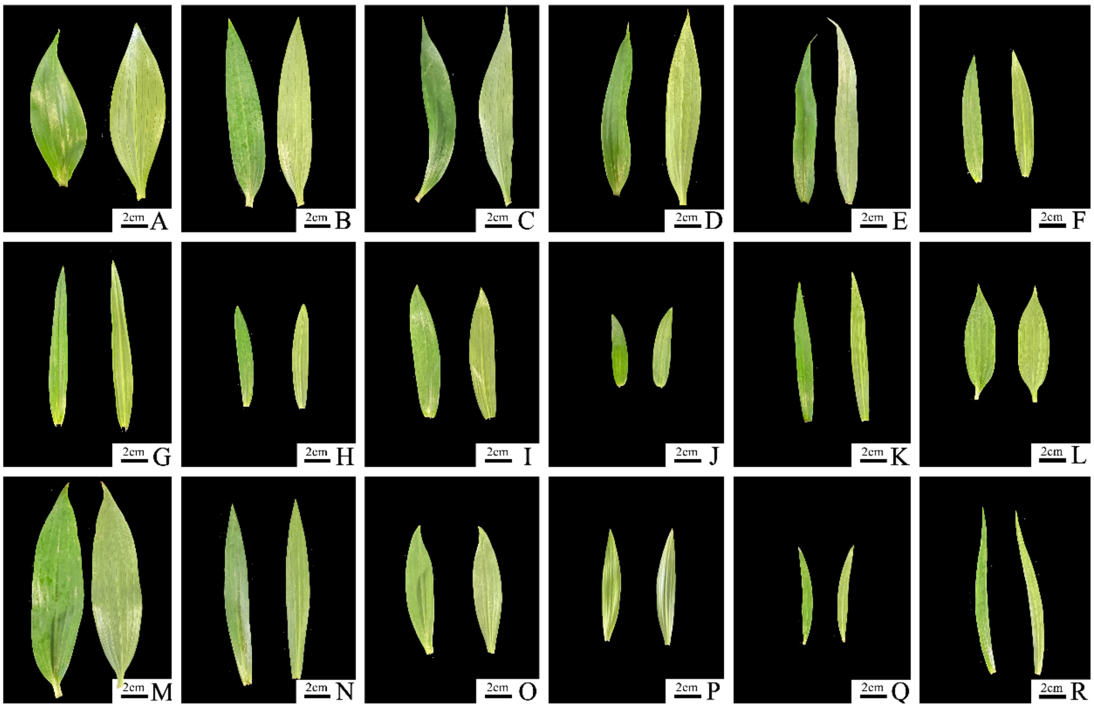

Figure S3. Leaf adaxial surface and abaxial surface of eighteen lilies. (A) 'Black Beauty'; (B) 'Conca D'or'; (C) 'Palazzo'; (D) 'Nymph'; (E) 'Friso'; (F) 'Eyeliner'; (G) 'Armandale'; (H) 'Heartstrings'; (I) 'Apricot Fudge'; (J) 'Trendy Havana'; (K) 'Secret Kiss'; (L) 'Cameleon'; (M) 'The Edge'; (N) 'White Triump'; (O) 'Magnefique'; (P) 'Watch Up'; (Q) *Lilium leucanthum*; (R) *Lilium lancifolium*.

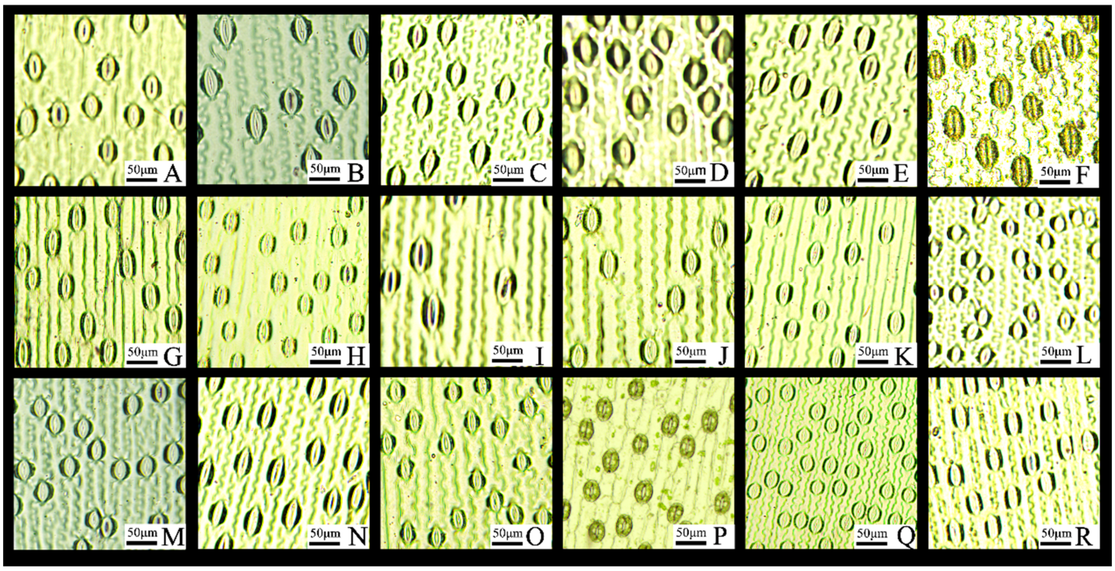

Figure S4. Leaf abaxial surface stomata of eighteen lilies. (A) 'Black Beauty'; (B) 'Conca D'or'; (C) 'Palazzo'; (D) 'Nymph'; (E) 'Friso'; (F) 'Eyeliner'; (G) 'Armandale'; (H) 'Heartstrings'; (I) 'Apricot Fudge'; (J) 'Trendy Havana'; (K) 'Secret Kiss'; (L) 'Cameleon'; (M) 'The Edge'; (N) 'White Triump'; (O) 'Magnefique'; (P) 'Watch Up'; (Q) *Lilium leucanthum*; (R) *Lilium lancifolium*.

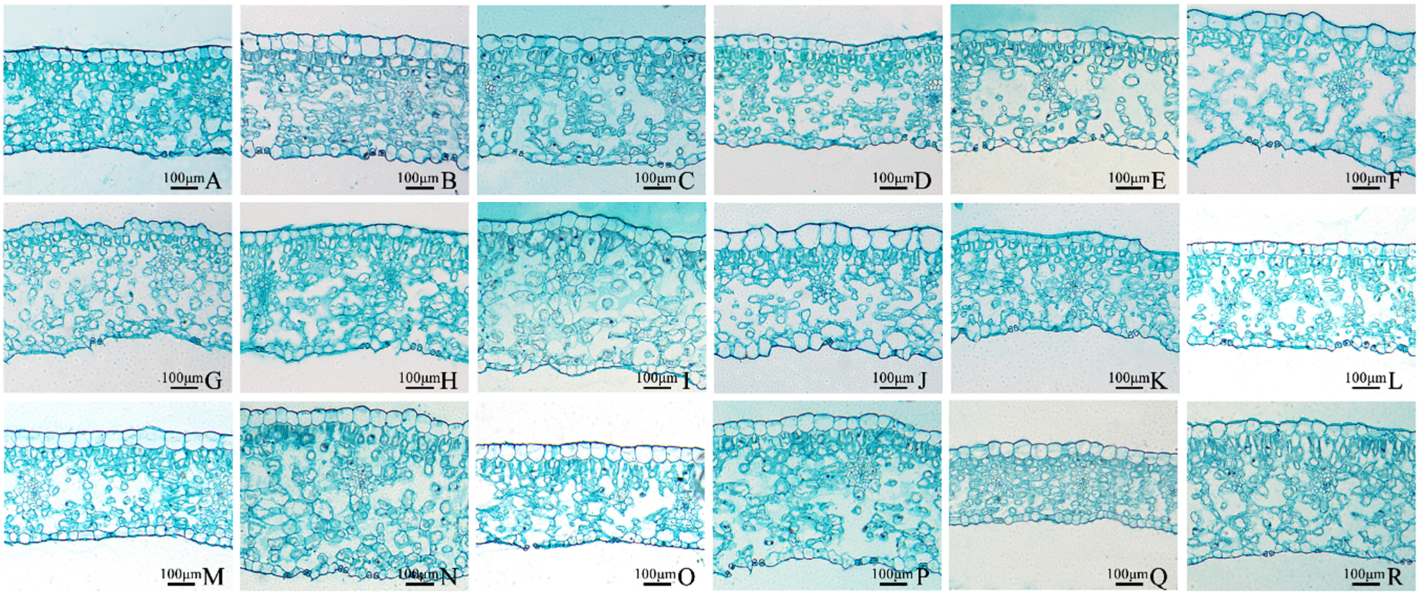

Figure S5. Leaf transverse sections of eighteen lilies, showing the epidermal tissues, and the palisade and spongy tissues. (A) 'Black Beauty'; (B) 'Conca D'or'; (C) 'Palazzo'; (D) 'Nymph'; (E) 'Friso'; (F) 'Eyeliner'; (G) 'Armandale'; (H) 'Heartstrings'; (I) 'Apricot Fudge'; (J) 'Trendy Havana'; (K) 'Secret Kiss'; (L) 'Cameleon'; (M) 'The Edge'; (N) 'White Triump'; (O) 'Magnefique'; (P) 'Watch Up'; (Q) *Lilium leucanthum*; (R) *Lilium lancifolium*.
